# Cross-cultural adaptation and psychometric properties of the Spanish Quality in Psychiatric Care Forensic Inpatient Staff (QPC-FIPS) instrument

**DOI:** 10.1038/s41598-022-17422-6

**Published:** 2022-08-03

**Authors:** Marta Domínguez del Campo, Antonio R. Moreno-Poyato, Montserrat Puig-Llobet, Maria Teresa Lluch-Canut, Nathalia Rodríguez Zunino, Manuel Tomás-Jiménez, Sara Sanchez-Balcells, Agneta Schröder, Lars-Olov Lundqvist, Gemma Escuder-Romeva, Juan Roldán-Merino

**Affiliations:** 1 Parc Sanitari Sant Joan de Déu, Dr. Antoni Pujades 42, 08830 Sant Boi de Llobregat, Spain; 2 Etiopatogenia I Tractament Dels Trastorns Mental Severs (MERITT), Institut de Recerca Sant Joan de Déu, Santa Rosa 39-57, 08950 Esplugues de Llobregat, Spain; 3 Fundació Privada Per La Recerca Sant Joan de Déu, Santa Rosa 39-57, 08950 Esplugues del Llobregat, Spain; 4 Public Health, Mental Health and Maternal-Infant Nursing Department, Nursing College, Universitat de Barcelona, Health Sciences Campus Bellvitge, Hospitalet de Llobregat, Barcelona, Spain; 5 Research Group GEIMAC (Consolidated Group 2017-1681: Group of Studies of Invarianza of the, Instruments of Measurement and Analysis of Change in the Social and Health Areas), Barcelona, Spain; 6 University Health Care Research Center, Faculty of Medicine and Health, Örebro University, Örebro, Sweden; 7 Department of Nursing, Faculty of Health Care and Nursing, Norwegian University of Science and Technology (NTNU), Trondheim, Norway; 8 Campus Docent Sant Joan de Déu, Miret i Sans, 10-16, 08034 Barcelona, Spain; 9 Research Group GIES (Grupo de Investigación en Enfermería, Educación y Sociedad), Barcelona, Spain

**Keywords:** Health care, Outcomes research

## Abstract

"Quality in Psychiatric Care-Forensic Inpatient Staff (QPC-FIPS) is an instrument of Swedish origin validated to measure the perception of the quality of mental health care provided by forensic psychiatry professionals. The aim of this study was to cross-culturally adapt the QPC-FIPS instrument and to evaluate the psychometric properties of the Spanish version of the instrument. A psychometric study was carried out. For validity, content validity, convergent validity and construct validity were included. For reliability, the analysis of internal consistency and temporal stability was included. The sample consisted of 153 mental health professionals from four Forensic Psychiatry units. The adapted Spanish version of the QPC-FIPS scale was configured with the same number of items and dimensions as the original. The psychometric properties, in terms of temporal stability and internal consistency, were adequate and the factor structure, such as the homogeneity of the dimensions of the Spanish version of the QPC-FIPS, was equivalent to the original Swedish version. We found that the QPC_FIPS-Spanish is a valid, reliable and easy-to-apply instrument for assessing the self-perception of professionals regarding the care they provide.

## Introduction

Several studies indicate that the forensic environment currently holds more people with mental health problems than any other institution; between 15 and 26% of people who have been in prison have been diagnosed with a mental health problem^1^. Another study indicates that one in seven people admitted to prison worldwide has suffered from a serious mental disorder^2^.

In Spain, the magnitude of this phenomenon has been described on several occasions. The most representative results were those from the study “Prevalence of mental disorders in Spanish prisons”. These results show that the prevalence mental health problem in a penitentiary environment is 84.4%^3^. To this, Tort et al. indicate that we must add the "factors related to one's own incarceration that have an influence on their health situation."^4^.

Consequently, there is a clear need to evaluate the quality of medical care provided by the professionals in penitentiary psychiatric units. In forensic psychiatry there is a growing interest in the evaluation of quality of life and care as an outcome measure^5^ and also as a variable in the evaluation of the likelihood of criminal recidivism^6^.

'Quality of medical care' was defined by Donabedian as *"*that which is expected to provide the user with the maximum and most complete well-being after assessing the balance of gains and losses that can accompany all its parts*"*^7^. Quality of medical care can be understood as a multidimensional concept based on three interrelated elements: professional care, interpersonal relationships and comforts of the environment^8^. One element to be taken into account in relation to the perception of the quality of medical care is job satisfaction. Numerous studies have emphasized the importance of this relationship^9^.The NTP 394 general satisfaction scale: Overall Job Satisfaction created by Warr^10^ is the most widely used in Spain and subsequently validated in Spanish by Pérez and Fidalgo^11^.

Measuring and improving the quality of health system care is of global interest^12^. Despite this, there is a lack of cross-cultural comparative studies on the perception of patients and professionals regarding quality of care^13,14^ and even fewer in the forensic field. This is mainly due to an absence of standardised psychiatric instruments for the measurement of medical care.

A systematic psychometric review was published in 2018^14^ in which 22 instruments on quality and satisfaction with mental health care according to professionals and patients were analyzed. This study shows that the instruments with the best psychometric properties most appropriate for the analysis of quality of care are: The Psychiatric Out-Patient Experiences Questionnaire (POPEQ)^15^, The Questionnaire of Experiences of General Practitioners (GPEQ)^16^, the Quality Indicator for Rehabilitative Care (QuIRC)^17^, Combined Assessment of Psychiatric Environments (CAPE)^18^ and the Quality in Psychiatric Care-Forensic In-Patient (QPC-FIP)^19^. The systematic review emphasises that the QPC instrument is the most suitable instrument for the measurement of quality of care in comparison with the other instruments mentioned above, as each of these instruments is specific to a given setting and population. In contrast, the QPC has been validated in both professional and patient versions and in different settings (community, hospital and forensic), allowing for a comprehensive assessment of quality of care and comparison of quality perception according to professionals and patients.

The QPC instrument was developed from the phenomenographic study by Schröder, Ahlström and Larsso^20^ which focused on the search for the dimensions that characterize the quality of care received in various areas of mental health care: hospital admission, community care and forensic psychiatry units. Each of the areas has developed a specific, self-administered instrument to assess quality of care from the perspective of the professionals and the people served^20^.

In Spain, the respective instruments for hospital professionals^21^, inpatients^22^ and outpatient staff^23^ have been validated. Within the scope that concerns us, the QPC-FIPS (Quality of Psychiatric Care-Forensic Inpatient Staff), QPC version for professionals in the field of forensic psychiatry, has not been validated in Spanish.

The current version of the QPC-FIPS scale is Swedish^24^ and the first cross-cultural adaptation is a Danish version^25^. Thus, it is considered necessary to carry out this adaptation to assess the quality of care provided in prison mental health units with the aim of improving such care and contributing to the comparison of results in different countries.

In this context, the objective of this study was to adapt the QPC-FIPS instrument to Spanish and analyze its reliability and validity.

## Methods

### Participants

The sample consisted of professionals from different disciplines who work in forensic mental health units (nursing, psychiatry, psychology, social education, nursing assistance, social work, and occupational therapy), who participate voluntarily. Having less than six month’s experience in the area of mental health was established as an exclusion criterion. Non-probability convenience sampling was used.

A total of 153 professionals participated, of whom 61.4% were women and 38.6% were men. The mean age was 39.92 ± 9.83 years, while the mean number of years worked in forensic units was 7.10 ± 4.94 years. The sample consisted of a variety of professional categories, with 34.6% nursing assistants, 22.9% occupational therapists, 21.6% nurses, 9.2% psychologists, 7.8% psychiatrists, and 3.9% social workers. Some 85% of the professionals stated that they would define their working environment as good or very good and 15% as bad or very bad. A total of 41.2% stated that they often or always have the opportunity to work on improvement of quality of medical care, 51.0% sometimes and 7.8% rarely or never.

### Instruments

Professional perception of quality care was obtained using QPC-FIPS-Spanish. This version is an adaptation of original questionnaire QPC-FIPS^24^.

The Quality of Psychiatric Care-Forensic Inpatient Staff (QPC-FIPS)^24^, described in the introduction section, is a self-administered questionnaire consisting of 34 items and measures 7 dimensions of quality of care: encounter (8 items), participation (8 items), discharge (3 items), support (4 items), Secluded environment (2 items), secure environment (3 items) and forensic specific (6 items). Each item is related to the statement "I consider that…" and responses are based on 4-point Likert scales, where 1 corresponds to "strongly disagree" and 4 to "strongly agree". All items also have the option "does not correspond".

To analyze convergent validity, the NTP 394 general satisfaction scale: Overall Job Satisfaction created by Warr et al.^10^ was used. This scale, which evaluates working conditions (intrinsic and extrinsic), consists of 15 items evaluated on a scale of 1 to 7 (1 “very dissatisfied” and 7 “very satisfied”)^11^.

### Procedure

The translation and back-translation process was carried out following the Standards for Educational and Psychological Testing^26^.

First, the original version was translated into Spanish by two independent native-speaker translators who had no knowledge of the instrument or the aims of the study. A group of experts comprising a specialist in psychometrics, three quality experts, five nurses specializing in mental health, a psychologist and a psychiatrist specializing in forensic medicine previously assessed semantic equivalence (grammatical difficulties in translation, equivalent meaning of words), and idiomatic (contextualization of the text, colloquialisms) and conceptual equivalence. The translation and back-translation process did not present any major difficulties. After the back-translation process, the expert committee suggested modifying some terms in items 30 and 33 to adapt them to the Spanish context, respecting the semantic meaning of the original version. Item 30 after back-translation of the Swedish version indicated: "the staff helps patients, if they wish, to present their wishes and their case before the administrative court"^24^, it was considered appropriate to modify it in the Spanish version to "users can access the judge through their lawyer or through the legal advice service”, changing the term "patients" to "users” since in Spain this term is widely used. Item 33 in the Swedish version indicated: "patients receive help from staff to elaborate their offence" for "during the stay in the prison the professionals help the user to talk about the offence he/she committed"^24^, so the term "patients" was replaced by "users" and "staff" by "professionals". Also "talking about the offence" was chosen instead of "elaborating the offence"^24^. Finally, inclusive non-discriminatory language was used when referring to gender.

A pilot cognitive test was administered to 30 professionals to assess comprehension and completion time. The test included an open-ended question asking participants to indicate whether any item might present difficulties in comprehension. The average completion time of the questionnaire ranged between 15 and 20 min and no item presented comprehension difficulties. Following the debriefing, it was not considered necessary to make any changes to either format of content. The Spanish adapted version of the QPC-FIPS scale was configured by the same number of items and dimensions as the original, with the final version in Spanish named QPC_FIPS-Spain.

The adaptation and psychometric evaluation process was carried out in four forensic mental health units at Parc Sanitari Sant Joan de Déu, Barcelona, Spain. Data collection was carried out between February, 2019 and December, 2021.

To analyze temporal stability, it was estimated that a minimum of 60 professionals would be needed to detect an intraclass correlation coefficient (ICC) around 0.70 between two administrations of the instrument, assuming a confidence level of 95% and a power of 80% in a bilateral comparison^22^.

### Data analysis

The SPSS Statistics program version 26 was used for analyses, along with EQS program version 6.2 for the confirmatory factor analysis (CFA)^27^.

Construct validity was analyzed through confirmatory factor analysis (CFA) with estimated parameters using the method of least squares (LS) which is similar to maximum-likelihood (ML) but estimates patterns of relationships between variables by minimising the sum of squares of the deviation between the hypothesised and observed model. LS performs better than ML with small samples and provides a better estimate when there is a violation of normal distribution assumptions^28^. The criteria for a good fit were Bentler Bonnet Normed Fit Index (BBNFI), Bentler Bonnet Non-Normed Fit Index (BBNNFI), Goodness of Fit Index (GFI), Adjusted Goodness of Fit Index (AGFI), Comparative Fit Index (CFI) > 0.90^29–31^; the ratio of chi square to degrees of freedom (χ2/df) < 3^32^. The value of the Root Mean Square Error of Approximation (RMSEA) and the Standarized Root Mean-Square (SRMR) and Root mean-square residual (RMR) was ≤ 0.08^33,34^.

The General Satisfaction Scale NTP 394: Overall Job Satisfaction was used for convergent validity^11^; an analysis of the Spearman’s correlation coefficient was performed. Furthermore, as an additional method, a Spearman’s correlation analysis was also performed between the factors of the QPC-FIPS instrument with the aim of verifying the Fayers & Machin^35^ hypothesis which indicates that the correlation was higher between each factor and the general scale than the correlations between the subscales.

To evaluate the internal consistency of the instrument at a general level and for each of the factors, Cronbach's alpha was used and ordinal Alpha, with a value greater than or equal to 0.70^36^ considered adequate reliability. Temporal stability or test–retest reliability was evaluated after 7–14 days through the ICC in a sample of 77 professionals. A value greater than or equal to 0.70 was considered an indicator of good agreement^36^. In addition, composite reliability was calculated. Item analyses included the calculation of item means, standard deviations, and corrected total item correlation.

### Ethical aspects of research

This study was approved by the research ethics committee at the Sant Joan de Déu Foundation, under CEIC code PIC-73-18. All research was conducted in accordance with relevant guidelines and regulations. All participants were informed about the objective of the study and gave their written consent to participate voluntarily and anonymously.

## Results

### Construct validity

Confirmatory factor analysis was used to verify the internal structure of the instrument. The CFA shows a chi-square value of (χ2 = 1115.705; df = 506; *p *< 0.0001). The GFI and AGFI show an adjustment > 0.90; the ratio between chi square and degrees of freedom (χ2/df) was 2.20, that is, achieving an index less than 3, and the value ≤ 0.08 was also obtained for the RMR, SRMR and RMSEA. All index showed a reasonable adjustment. Table 1 shows the fit of the model.

**Table 1:** Goodness-of-fit indices for the confirmatory model QPC-FIPS Spanish.

BBNFI	0.613
BBNNFI	0.709
GFI	0.935
AGFI	0.923
CFI	0.738
RMR	0.048
SRMR	0.086
RMSEA	0.089
Cronbach’s alpha	0.916
Goodness of fit test	χ2 = 1115.705; df = 506; p < 0.0001
Adjustment reason	χ2 / df = 2.20

*BBNFI*: Bentler bonnet normed fit index, *BBNNFI* Bentler Bonnet non-normed fit index, *GFI* Goodness of fit index, *AGFI* Adjusted goodness of fit index, *CFI* Comparative fit index, *RMR* Root mean-square residual, *SRMR* Standarized root mean-square, *RMSEA* Root mean square error of approximation, *df* Degrees of freedom.

**Figure 1:**
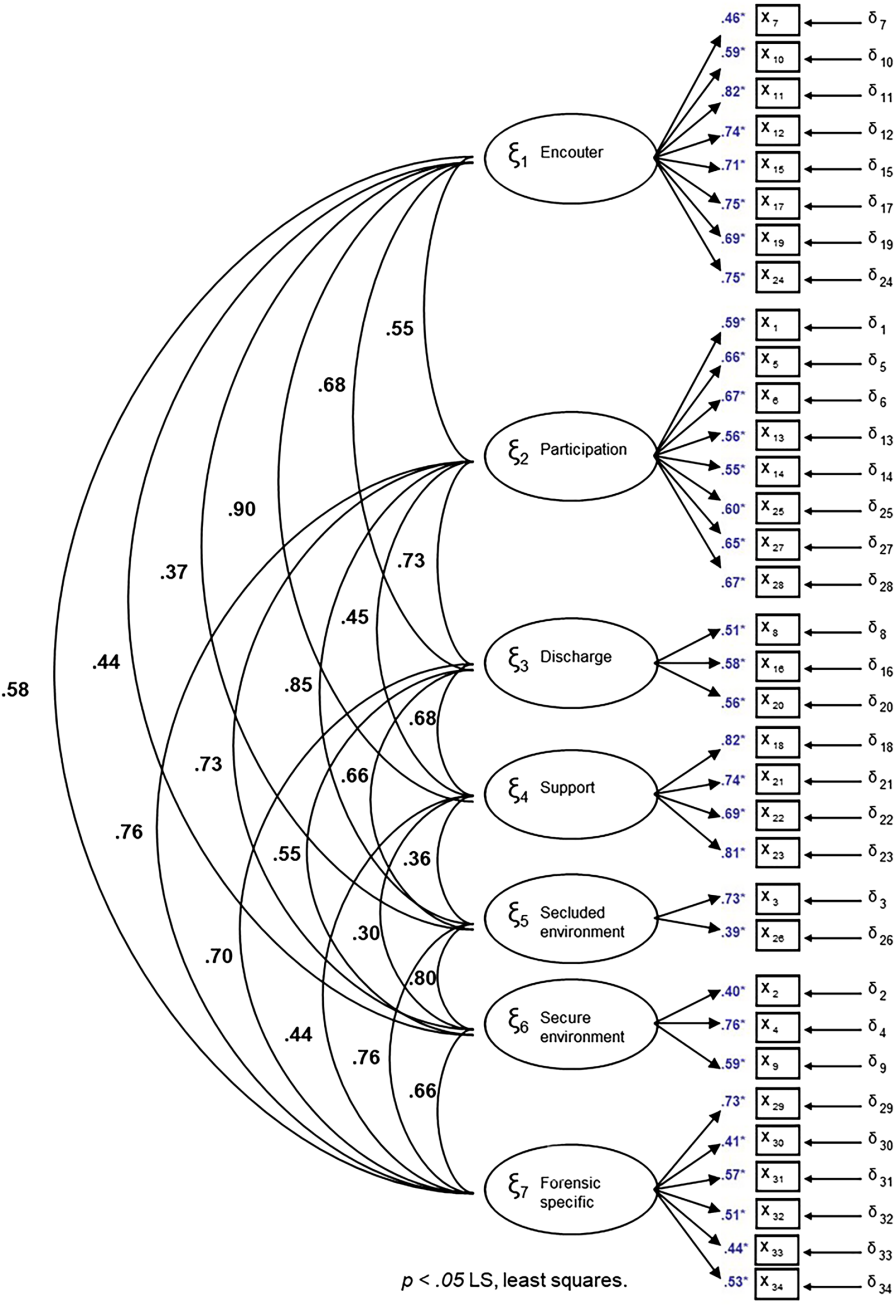
Factor loadings derived from the LS estimation (least squares).

The adaptation in Spanish reproduces the structure of the instrument in its original language where the different items are grouped into 7 factors. Figure 1 shows the saturation of all items. All saturations were equal to or greater than 0.40.

### Convergent validity

Convergent validity was analyzed using Spearman’s correlation coefficient with the NTP 394 general satisfaction scale: Overall Job Satisfaction^11^. The correlation obtained was rho = 0.438 (*p* < 0.0001). Table 2 shows the correlations between factors and with the total instrument score. F1."Encounter" and F2." Participation" correlated most strongly with the total instrument (rho = 0.886 and rho = 0.834 respectively).

**Table 2:** Correlations among the subscales of the SPANISH QPC-FIPS.

	Encounter	Participation	Discharge	Support	Secluded environment	Secure environment	Forensic specific	Total QPC-FIPS
F1. Encounter	1							
F2. Participation	0.691*	1						
F3. Discharge	0.608*	0.574*	1					
F4. Support	0.478*	0.494*	0.592*	1				
F5. Secluded Environment	0.436*	0.431*	0.432*	0.434*	1			
F6. Secure Environment	0.609*	0.606*	0.522*	0.415*	0.454*	1		
F7. Forensic specific	0.613*	0.491*	0.515*	0.433*	0.351*	0.481*	1	
Total QPC-FIPS	0.886*	0.834*	0.736*	0.662*	0.566*	0.732*	0.774*	1

*n* = 153.

*All correlation coefficients (rho) are significant at *p* ˂0.01.

### Reliability

Table 3 shows the description of each of the items. The mean value of the items ranged from 2.27 to 3.71 (standard deviation from 0.54 to 1.0). On the other hand, the corrected total item correlation was higher than 0.20 in all items. Nor does instrument reliability improve if any of the items are eliminated.

**Table 3:** Descriptive Statistics of the Items of the QPC-FIPS Scale and Cronbach’s alpha.

Summary of the contents of the items		Mean	SD	Corrected item-total correlation	Cronbach’s alpha Total instrument without item
*Encounter*		11.50	3.86		
P7	Gives support when the users need it	3.48	0.73	0.349	0.915
P10	Committed professionals	3.34	0.83	0.509	0.913
P11	Shows empathy	3.57	0.64	0.661	0.912
P12	Cares if the users get angry	3.66	0.62	0.568	0.913
P15	Respects the users	3.61	0.62	0.526	0.913
P17	Shows understanding	3.26	0.72	0.622	0.912
P19	Has time to listen	3.56	0.65	0.533	0.913
P24	Cares about the users’ care	3.66	0.57	0.553	0.913
*Participation*		16.13	4.37		
P1	Users have influence over their care	2.27	0.81	0.460	0.914
P5	Users’ view of the right care is respected	2.75	0.78	0.573	0.913
P6	Users take part in decision-making about their care	2.59	0.81	0.562	0.913
P13	Benefit drawn from the patient’s earlier experience of treatment	2.99	0.83	0.480	0.914
P14	Users helped to recognize signs of deterioration	3.39	0.78	0.492	0.914
P25	Users informed in a way that they understand	3.20	0.71	0.474	0.914
P27	Users have knowledge about their mental troubles	3.11	0.69	0.548	0.913
P28	Users receive information about treatment alternatives	2.66	0.81	0.558	0.913
*Discharge*		5.63	1.85		
P8	Planning of the users’ continued treatment	2.98	0.76	0.433	0.914
P16	Users are offered follow-up after discharge	2.84	1.08	0.474	0.914
P20	Users know where to turn	3.45	0.72	0.460	0.914
*Support*		5.25	2.02		
P18	Stops the users from hurting others	3.64	0.59	0.573	0.913
P21	Stops the users from hurting themselves	3.71	0.54	0.481	0.914
P22	Nothing shameful about having mental troubles	3.69	0.57	0.518	0.914
P23	Shame and guilt must not get in the way	3.71	0.55	0.590	0.913
*Secluded environment*		3.72	1.31		
P3	Access to secluded place	2.92	0.94	0.471	0.914
P26	There’s a secluded place	3.06	0.92	0.276	0.917
*Secure environment*		6.02	1.77		
P2	High level of security in ward	3.22	0.84	0.275	0.917
P4	Feel secure with fellow users	2.99	0.76	0.526	0.913
P9	Not disturbed by fellow users	2.38	0.78	0.370	0.915
*Forensic specific*		12.51	3.06		
P29	Informed of their rights	2.97	0.84	0.604	0.912
P30	Help the users in contact with the Administrative Court	3.45	0.84	0.290	0.917
P31	The doctor explains the users’ medical reports clearly	2.79	0.82	0.465	0.914
P32	Support from their lawyer	2.84	0.76	0.410	0.915
P33	Professionals help the users to talk about their crimes	2.94	0.78	0.362	0.915
P34	Professionals involved in the’ users’ care	2.56	0.77	0.414	0.915

*SD* Standard deviation.

Cronbach's alpha of the internal consistency coefficient of the total instrument scored high. However, it should be noted that three of the seven factors (F3.“Discharge”, F5.“Secluded Environment” and “F6.Secure Enviroment”) obtained lower values. Table 4 shows the results.

**Table 4:** Spanish QPC-FIPS.

Factors	ICC (CI 95%)	Cronbach’s alpha	Alpha ordinal
F1. Encounter	0.641 (0.435–0.771)	0.864	0.872
F2. Participation	0.903 (0.847–0.938)	0.823	0.832
F3. Discharge	0.865 (0.788–0.914)	0.558	0.565
F4. Support	0.605 (0.379–0.749)	0.853	0.849
F5. Secluded Environment	0.867 (0.790–0.915)	0.405	0.443
F6. Secure Environment	0.877 (0.807–0.922)	0.590	0.596
F7. Forensic specific	0.732 (0.578–0.830)	0.698	0.701
Total	0.802 (0.689–0.874)	0.916	0.956

Test–retest ICC (n = 77) and Cronbach’s alpha.

*ICC* Intraclass correlation coefficient, *CI* Confidence interval.

The ICC analysis showed that the test–retest reliability was 0.802 (95% CI: 0.689–0.874; n = 77), and this value was greater than. 70 in all instrument factors except F1."Encounter" and F4." Support" with values of 0.641 and 0.605, respectively (Table 4).

## Discussion

The objective of this study was to adapt the Quality in Psychiatric Care Inpatient Staff (QPC-FIPS) instrument to Spanish, as well as analyzing its reliability and validity.

The process of translation and back-translation from the original version^24^ did not present any major difficulties. The expert committee suggested modifying two elements to improve comprehension in their cross-cultural adaptation. These results are consistent with what is specified in the Danish adaptation^25^ of the instrument, therefore, it is demonstrated that the construct of quality in care in penitentiary mental health units has a similar meaning to that proposed in the Swedish or Danish context.

In relation to the sample used, it is worth noting the smaller number of participants (153) in our study compared to the original Swedish version (348)^24^ and the Danish adaptation (630)^25^; although the percentage proportion of women (60%) and men (40%) with a similar mean age and representation of the different professional categories in a similar way is maintained. It should be noted that for the Spanish adaptation of the QPC-IPS instrument the sample was similar (163) and adequate psychometric properties were also obtained^21^.

At the psychometric level, the results of the Spanish adaptation of the QPC-FIPS were adequate. Regarding construct validity, the CFA showed a significant chi-square value (χ2 = 1115.705; df = 506; *p* < 0.0001); It should be noted that the GFI and AGFI values were ≥ 0.90. The ratio between chi square and degrees of freedom (χ2/df) was ≤ 3. The RMSEA, SRMR and RMR index values indicated values ≤ 0.08. results which are very similar to the Danish^25^ and Swedish versions^24^ of QPC-FIPS and Spanish adaptation of QPC-OPS^23^ and QPC-IPS^21^.

The convergent validity of the instrument was calculated using the Spearman’s correlation ratio between the QPC-FIPS and the NTP 394 general satisfaction scale: Overall Job Satisfaction. The correlation obtained was positive. These data were similar to those reported in the results of the QPC-OPS (rho = 0.31)^23^ and QPC-IPS (rho = 0.58)^21^ validation studies. Furthermore, as an additional method, a Spearman’s correlation analysis was also performed between the factors of the QPC-FIPS questionnaire. It was observed that, in the vast majority of cases, the correlation coefficients between the QPC-FIPS factors were moderate (0.40–0.60). While it is true that a greater degree of correlation between the correlation coefficients is observed in the Danish^25^ or Swedish version^24^, we noted that the correlation was higher between each factor and the general scale than the correlations between the subscales, confirming the Fayer and Machin hypothesis^35^.

With respect to reliability, the internal consistency of the instrument was evaluated at a general level, showing a Cronbach's alpha of 0.91, which is similar to that obtained with the original version (0.94)^24^, the Danish adaptation (0.93)^25^. and of the Spanish adaptations of the QPC-IPS (0.92)^21^ and QPC-OPS(0.88)^23^. Regarding the internal consistency of each of the factors, it should be noted that in each of the versions of the QPC-FIPS created so far, Swedish, Danish and Spanish, the factors with the lowest score are F3. "Discharge", F5. "Secluded environment" and F6. "Secure Environment"; with F7 "Forensic specific"^24^ added to this list from the Danish version^25^. The ICC analysis showed that the test–retest reliability was adequate at 0.80 (95% CI: 0.689–0.874; n = 77). These results were similar to those reported in validations of QPC versions that have reported the ICC: QPC-IPS 0.91 (95% CI: 0.87–0.94; n = 92)^21^ and QPC-OPS 0.84 (95% CI: 0.790–0.888; n = 157)^23^.

When analyzing the psychometric measures of the QPC-IPS-Spanish scale, validated in the hospital context, we found that the factors indicated above: F3. "Discharge", F5. "Secluded environment" and F6. "Secure Environment" also show a lower score^21^. Finally, the psychometric properties of this instrument also agree with those obtained in the adaptation of QPC_OPS-Spanish scale, validated at the community level^23^.

## Limitations

One limitation was the sample size used in this study. Since the sample is a factor that interacts with aspects of the design such as the communality of the data and the number of variables per factor, following the recommendations a sample size of 200 subjects is required when we have communalities between 0.40 and 0.70, and the number of variables per factor is 3–4 items^37^. In this study, the sample was slightly less than 200 so the data should be interpreted with caution. However, recruitment itself was a limitation due to the limited number of professionals working in the field of forensic psychiatry. However, this limitation is mitigated by the comparison of similar psychometric outcomes in the original studies^24^ as well as those carried out in our context with mental health professionals from other healthcare settings^21^.

In terms of convergent validity, one limitation is the use of NTP 394 general satisfaction scale: Overall Job Satisfaction^11^. This is a questionnaire that allows us to assess overall job satisfaction. It may be that this instrument is not the most suitable for analysing this validity, but it has been translated and validated in Spanish and is one of the most widely used instruments. It was selected because although the two instruments do not assess exactly the same thing, the literature indicates that professionals who report higher perceptions of quality of care are those who report higher job satisfaction^9^.

Steps should be taken in future research to avoid the present limitations.

## Conclusion

The results obtained indicate that the psychometric properties in terms of temporal stability and internal consistency are adequate and that the factorial structure of the Spanish version of the QPC-FIPS is equivalent to the original Swedish version^24^, demonstrating that the concept of quality perceived by professionals at forensic psychiatry units in Spain fits the concept of their counterparts in Sweden^24^. Likewise, the factorial loads, such as the homogeneity of the dimensions, were also similar to those of the Swedish version. The Spanish version of QPC-FIPS is the first instrument that allows assessment of the perceptions of forensic psychiatry professionals in Spain regarding the care they provide.

## Data Availability

The data that support the findings of this study are available upon reasonable request from the corresponding author. The data are not publicly available due to privacy and ethical restrictions. The data were taken from our own study.
